# Supplementary material for: Integrated Discriminant Evaluation of Molecular Genetic Markers and Genetic Diversity Parameters of Endangered Balearic Dog Breeds
Source: Int J Mol Sci. 2024 Feb 26;25(5):2706. doi: 10.3390/ijms25052706 (PMC10932221; doi:10.3390/ijms25052706)
Supplement: Supplementary file 1 [file Supplementary Figure S1.pdf › Supplementary Figure S1.pdf]

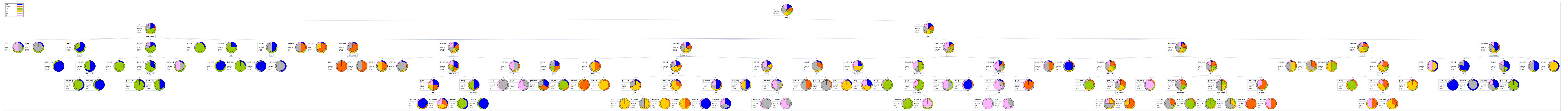

Figure S1. CHAID decision tree representing the patterns that genetic diversity parameters and molecular microsatellite markers follow across the different breeds in the Balearic Archipelago.
